# Employees and the National Insurance Act

**Published:** 1912-10-05

**Authors:** 


### NATIONAL INSURANCE ACT DEPARTMENT.

### EMPLOYEES AND THE NATIONAL INSURANCE ACT.

### XXIX.—The Approaching End of the First Quarter.

On October 14 the National Insurance Act will have been in operation for three months, and this date marks an important epoch in the system of National Insurance. Not only is it the date on which the second insurance cards will become current, but it is also the date by which, nominally at least, all persons who were employed on July 15 and who come within the compulsory provisions of the Act must have chosen whether they will obtain their benefits as deposit contributors or as members of approved societies. In view of the fast approaching close of the quarter we must make special reference to some important details connected with the sending in of the insurance cards now current, and we also feel it incumbent upon us, in the best interests of our readers who are insured persons, to reiterate the necessity for making a wise choice of an approved society. The latter subject is, perhaps, the more important, and will, accordingly, be dealt with first.

#### Disadvantages of Deposit Contributors.

It cannot be too strongly emphasised that by becoming a deposit contributor an insured person is relinquishing one of the principal advantages of the insurance scheme—namely, that of co-operation. The deposit contributor, in fact, does not obtain the advantages of insurance at all. He merely gets back, in the form of benefits, when he may be entitled to them, the money he himself has paid, together with the contributions of his employer and the State grant of two-ninths (or in the case of women one-quarter). Before any benefits can be paid to him his account is to be charged in advance with seven-ninths (or in the case of women three-quarters) of the costs of administration and of medical and sanatorium benefits for the ensuing year, and as soon as his own fund is exhausted the payment of benefits cease. Furthermore, those who enter insurance within one year of the commencement of the Act as members of approved societies above the age of sixteen bring with them as a credit to their society in the books of the Insurance Commissioners a certain reserve value to enable them to be accepted at the level rate of contribution irrespective of their age at entry. This credit of a reserve value is on the whole much more valuable than the State grant in aid of the benefits which it is replacing for a period estimated at about eighteen years. No such crediting of reserve values is required in the case of deposit contributors, and accordingly another disadvantage is added to those already mentioned. We have already referred to this matter more than once in previous insurance articles, but we have no apology to offer for this repeated reference in view of the paramount importance to those of our readers who are insured persons. If further justification were needed we have only to point out that the Insurance Commissioners felt it necessary to emphasise its importance by a double reference in their famous "N.H." Leaflet, which was so widely circulated just before the Act came into operation, in the words "*The full benefits of insurance can only be obtained by joining an approved society.*"

#### Insurance Cards obtained from Post Offices.

Many insured persons had not joined an approved society by July 15, and in consequence obtained their first insurance card from the Post Office. It does not follow, however, that they will have to become deposit contributors. It is only when the stamped card is returned to the Post Office that the entry of the contributions, represented by the stamps, is made in the Post Office fund, and the person becomes a deposit contributor. A card obtained from the Post Office during the current quarter can be returned to an approved society, provided application for membership has already been accepted by the society, just as though the card had originally been issued by the society.

Every insured person who has obtained his card from a Post Office has, therefore, nominally another week only in which to select his approved society, if he has not already exercised his choice. It is possible, however, for the period to be extended by a few extra days, in case of need, because the time allowed to the approved societies, under the instructions of the Commissioners, for the collection of the cards at the end of the quarter is a fortnight dating from October 14.

Delay is always dangerous, and never more so than in matters connected with insurance, and our advice to those who have deferred applying to an approved society for membership is to "do it now."

There is really no excuse for further delay. So many societies have now obtained the approval of the Commissioners that an insured person who cannot find one to suit his requirements must indeed be hard to please. A full list of societies that have been approved, which sets forth in addition to the name of the society the name of the secretary and of the principal office, and whether the society is for men or women or for both sexes, and the countries of the Union in which its operations are conducted, may be obtained on application at any Post Office.

#### The Nurses' Insurance Society.

As so many of our readers are members of the nursing profession it is fitting that we should state explicitly that, in our opinion, the society best suited to their requirements as a class is the Nurses' Insurance Society, of which Mr. L. M. Dick is secretary, and whose offices are at 15 Buckingham Street, Strand, London, W.C. We have consistently recommended this society to correspondents, as will have been noticed from answers to inquiries in our columns. Our chief grounds for recommending the society are that it has been founded for the sole benefit of female trained nurses, and their interests as a class will, therefore, be specially studied; and, having the advantage of connection, as a separate section, with the Royal National Pension Fund for Nurses, it goes without saying that every detail will be managed with the same business ability and consideration for the members that have always characterised that Fund.

### Other Societies.

For other insured persons we have not recommended any particular society, and we do not intend to do so now. Those who were members of Friendly Societies prior to the commencement of the Act are probably wise in continuing their membership, if their society has become approved, for the purpose of the State insurance. The decision, of course, depends upon the particular circumstances of the individual society, and the aptitude its officials have shown in its administration in the past and in the adoption of the new order of things.

In the same way it is only to be expected that those who were members of trade unions or co-operative societies should favour membership in their existing society for the purposes of the Act, and the officials of the unions have shown a zeal in obtaining new members that is quite phenomenal. The approved societies formed in connection with or as separate sections of the large industrial insurance companies and collecting societies have, during the past few months, carried out an active propaganda in explaining the Act and in recruiting members for their societies. With their wide agency connections and perfect organisations these societies are in a particularly favourable position for administering the benefits of the Act, and the fact that they had obtained so many members, running into millions, as reported by the Chancellor of the Exchequer before the rising of Parliament, is of itself a full justification for allowing them to become approved. It is safe to say that without their aid, and the expert knowledge of organising huge numbers which they brought to bear on the problem, it would have been impossible to have put the machinery of the Act into operation on July 15.

### Sending in Insurance Cards.

The other matter to which we referred at the outset is the sending in of the current insurance cards and the issue in their place of the corresponding cards for the second quarter. This is a stupendous task to be accomplished within the fortnight prescribed by the Commissioners, and the co-operation of the insured persons must be obtained by the officials of the societies if it is to be carried out in such a short time. According to the regulations it is necessary for the insured person to send his current card to his society, or to the Post Office if he is a deposit contributor, but it is obviously impossible for such a proceeding to be carried out literally in view of the large numbers involved and the necessity for writing and the cost of postage that it would entail. Some of the smaller societies might with reason expect their members to comply with the regulation, but all the larger societies are making arrangements for the cards to be collected, and supplies of the new cards are already in hand for delivery in their place.

Before handing the card to the society's representative it should be signed by the member, and it is only after the stamps have been affixed and it has been returned to the member by his employer that it is legal to write thereon the name of the society or the membership number. Every member of an approved society should have been furnished with an insurance book. At the end of this book full particulars are given of the value of the stamps that should have been affixed to the card. In the normal case the matter is quite simple. That is to say, a 7d. stamp is required in the case of a man and a 6d. stamp in the case of a woman for each week during any part of which they have rendered services to an employer, or for which they have received any remuneration from him. Any variation of these rates, as for instance when low wages (not exceeding 2s. a day) are paid to persons over twenty-one years of age, and for whom board and lodging is not provided, must be authenticated by a declaration signed by the insured person specifying each week to which the variation applies. For the purpose of record the number of stamps affixed to the card, and a note of their value, must be entered in the member's insurance book by the society's official, and the entry acknowledged by his initial. In many cases, therefore, the insurance book will be collected as well as the card, but if this is done the member will have the right of demanding the return of the book within three weeks from the date of its collection. In other cases, the societies will arrange to allow their representatives to make the entry in the book at the time the card is collected, thus obviating a second call.

There is one further point that should be carefully noted, and that is that the employer is not required to affix a stamp when no services have been rendered and no remuneration paid for any given week. It is possible, therefore, that through unemployment cards may be handed in without their full complement of thirteen stamps. In such case the member will ordinarily be in arrear to the extent of the missing contributions. Three weeks a year on the average are allowed before arrears count. If they are not paid by the insured person himself he will suffer a reduction of benefit according to scale. For the first year of the operation of the Act arrears are not to count, and in consequence there will not be the same inducement to pay them up as will be the case later on. The only reason for paying up contributions falling due for weeks of unemployment within the first year is that the waiting periods that have to elapse before the benefits can be claimed depend in part upon the number of contributions actually paid.

**NOTE.—Questions and Correspondence on all doubtful points are invited, and should be addressed to the Editor, The Hospital, 28 Southampton Street, Strand, W.C., and marked "Insurance."**

